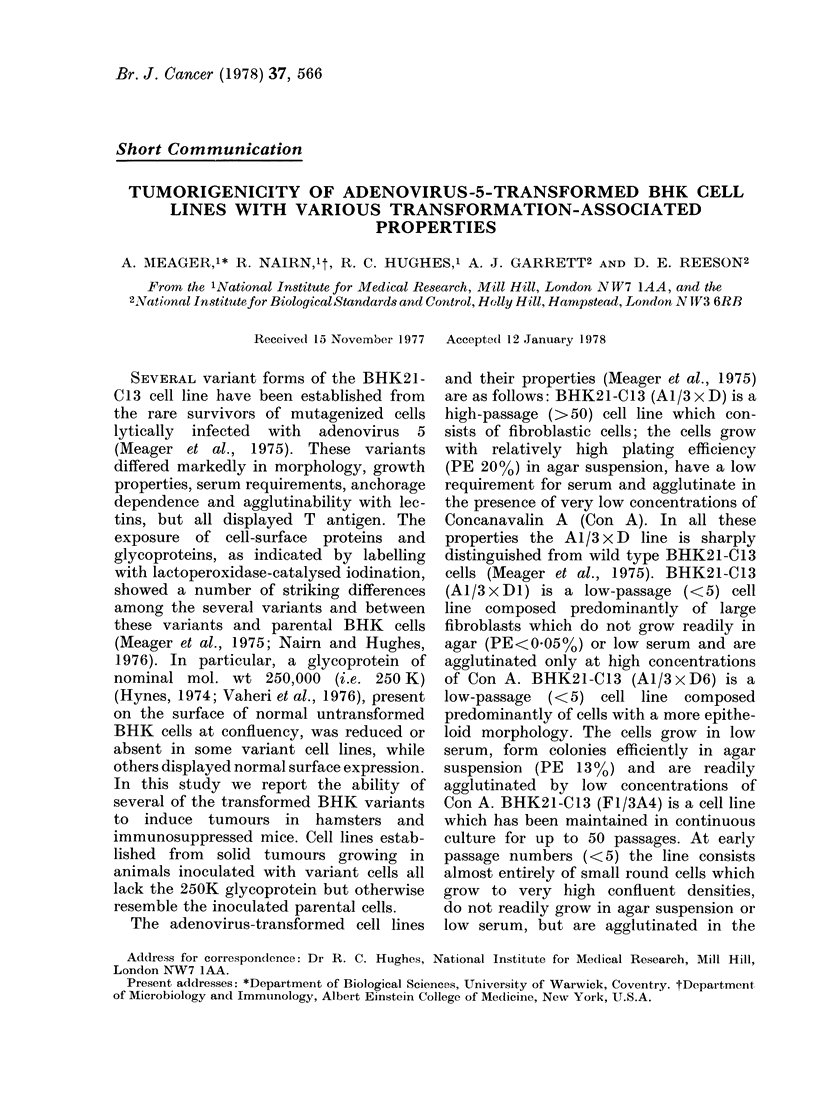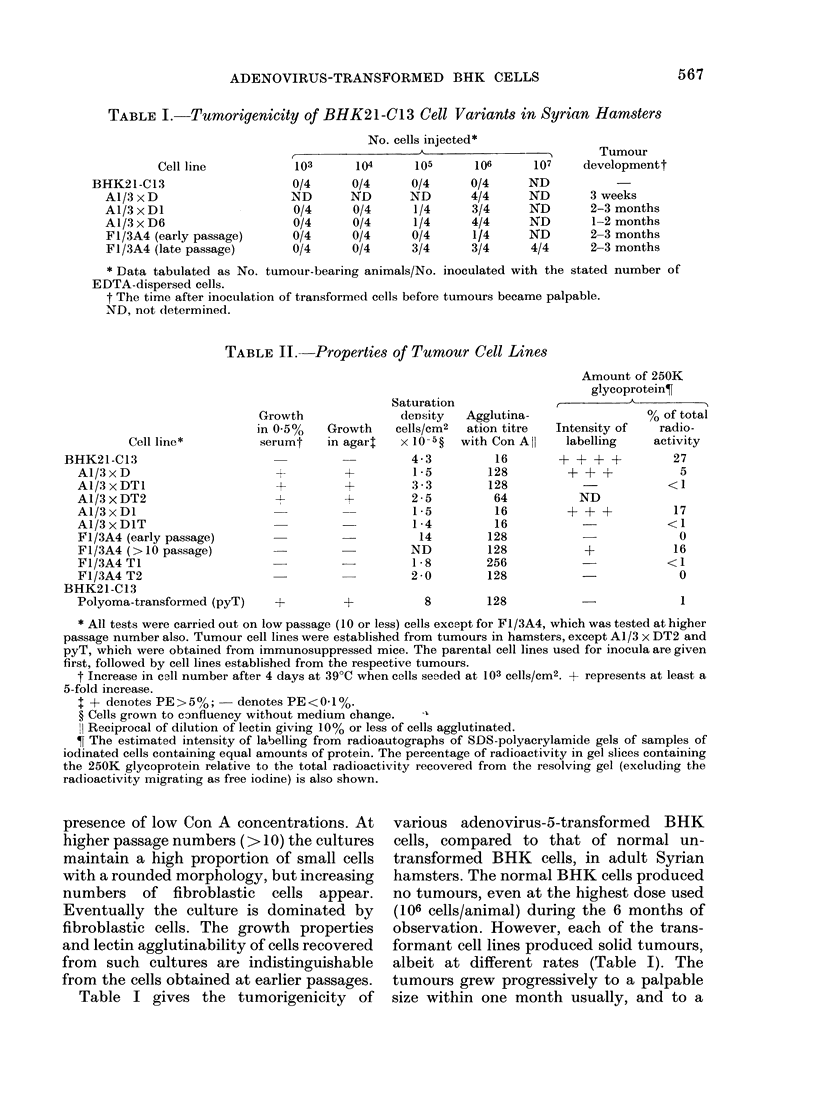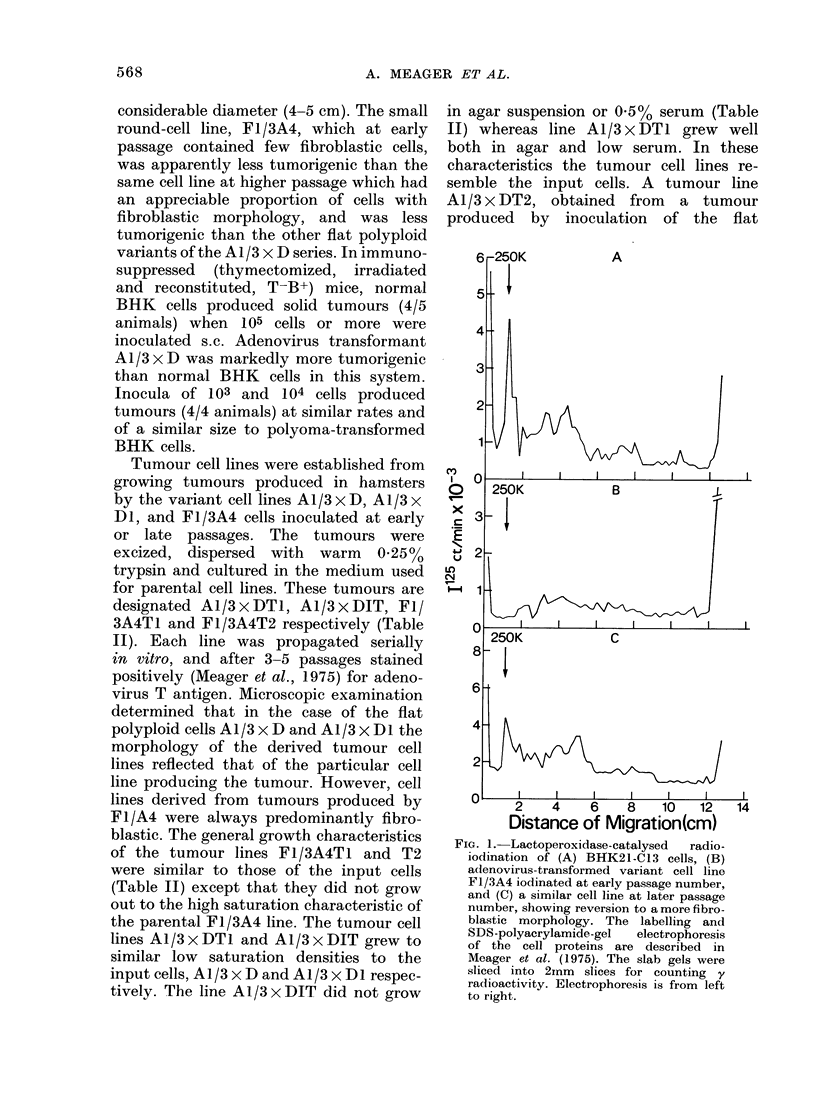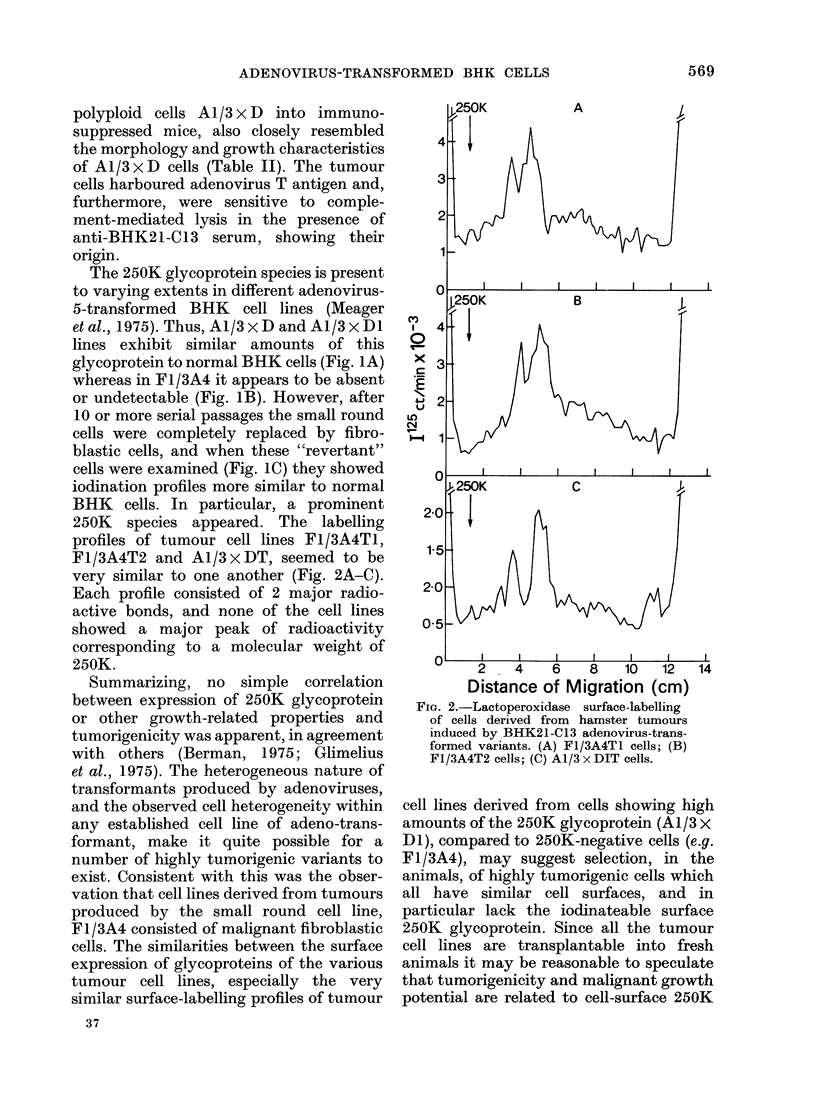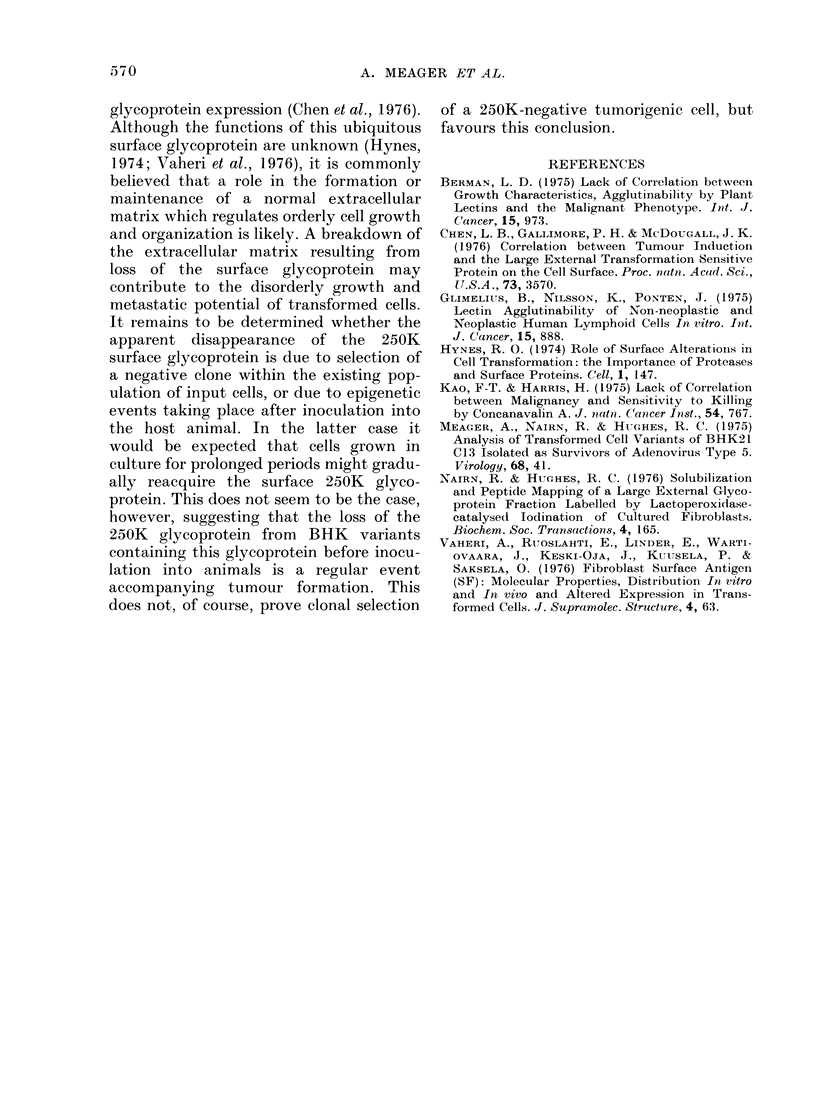# Tumorigenicity of adenovirus-5-transformed BHK cell lines with various transformation-associated properties.

**DOI:** 10.1038/bjc.1978.85

**Published:** 1978-04

**Authors:** A. Meager, R. Nairn, R. C. Hughes, A. J. Garrett, D. E. Reeson


*Br. J. Cancer* (1978) **37**, 566

### *Short Communication*

### TUMORIGENICITY OF ADENOVIRUS-5-TRANSFORMED BHK CELL LINES WITH VARIOUS TRANSFORMATION-ASSOCIATED PROPERTIES

A. MEAGER,[1*] R. NAIRN,[1†], R. C. HUGHES,[1] A. J. GARRETT[2] AND D. E. REESON[2]

*From the [1]National Institute for Medical Research, Mill Hill, London NW7 1AA, and the [2]National Institute for Biological Standards and Control, Holly Hill, Hampstead, London NW3 6RB*

Received 15 November 1977 Accepted 12 January 1978

SEVERAL variant forms of the BHK21-C13 cell line have been established from the rare survivors of mutagenized cells lytically infected with adenovirus 5 (Meager *et al.*, 1975). These variants differed markedly in morphology, growth properties, serum requirements, anchorage dependence and agglutinability with lectins, but all displayed T antigen. The exposure of cell-surface proteins and glycoproteins, as indicated by labelling with lactoperoxidase-catalysed iodination, showed a number of striking differences among the several variants and between these variants and parental BHK cells (Meager *et al.*, 1975; Nairn and Hughes, 1976). In particular, a glycoprotein of nominal mol. wt 250,000 (*i.e.* 250 K) (Hynes, 1974; Vaheri *et al.*, 1976), present on the surface of normal untransformed BHK cells at confluency, was reduced or absent in some variant cell lines, while others displayed normal surface expression. In this study we report the ability of several of the transformed BHK variants to induce tumours in hamsters and immunosuppressed mice. Cell lines established from solid tumours growing in animals inoculated with variant cells all lack the 250K glycoprotein but otherwise resemble the inoculated parental cells.

The adenovirus-transformed cell lines and their properties (Meager *et al.*, 1975) are as follows: BHK21-C13 (A1/3×D) is a high-passage (>50) cell line which consists of fibroblastic cells; the cells grow with relatively high plating efficiency (PE 20%) in agar suspension, have a low requirement for serum and agglutinate in the presence of very low concentrations of Concanavalin A (Con A). In all these properties the A1/3×D line is sharply distinguished from wild type BHK21-C13 cells (Meager *et al.*, 1975). BHK21-C13 (A1/3×D1) is a low-passage (<5) cell line composed predominantly of large fibroblasts which do not grow readily in agar (PE<0·05%) or low serum and are agglutinated only at high concentrations of Con A. BHK21-C13 (A1/3×D6) is a low-passage (<5) cell line composed predominantly of cells with a more epitheloid morphology. The cells grow in low serum, form colonies efficiently in agar suspension (PE 13%) and are readily agglutinated by low concentrations of Con A. BHK21-C13 (F1/3A4) is a cell line which has been maintained in continuous culture for up to 50 passages. At early passage numbers (<5) the line consists almost entirely of small round cells which grow to very high confluent densities, do not readily grow in agar suspension or low serum, but are agglutinated in the

Address for correspondence: Dr R. C. Hughes, National Institute for Medical Research, Mill Hill, London NW7 1AA.

Present addresses: *Department of Biological Sciences, University of Warwick, Coventry. †Department of Microbiology and Immunology, Albert Einstein College of Medicine, New York, U.S.A.

TABLE I.—*Tumorigenicity of BHK21-C13 Cell Variants in Syrian Hamsters*

| Cell line | No. cells injected* $10^3$ | $10^4$ | $10^5$ | $10^6$ | $10^7$ | Tumour development† |
|---|---|---|---|---|---|---|
| BHK21-C13 | 0/4 | 0/4 | 0/4 | 0/4 | ND | — |
| A1/3 × D | ND | ND | ND | 4/4 | ND | 3 weeks |
| A1/3 × D1 | 0/4 | 0/4 | 1/4 | 3/4 | ND | 2–3 months |
| A1/3 × D6 | 0/4 | 0/4 | 1/4 | 4/4 | ND | 1–2 months |
| F1/3A4 (early passage) | 0/4 | 0/4 | 0/4 | 1/4 | ND | 2–3 months |
| F1/3A4 (late passage) | 0/4 | 0/4 | 3/4 | 3/4 | 4/4 | 2–3 months |

* Data tabulated as No. tumour-bearing animals/No. inoculated with the stated number of EDTA-dispersed cells.

† The time after inoculation of transformed cells before tumours became palpable.

ND, not determined.

TABLE II.—*Properties of Tumour Cell Lines*

| Cell line* | Growth in 0·5% serum† | Growth in agar‡ | Saturation density cells/cm² × $10^{-5}$§ | Agglutination titre with Con A‖ | Amount of 250K glycoprotein¶ Intensity of labelling | % of total radioactivity |
|---|---|---|---|---|---|---|
| BHK21-C13 | — | — | 4·3 | 16 | + + + + | 27 |
| A1/3 × D | + | + | 1·5 | 128 | + + + | 5 |
| A1/3 × DT1 | + | + | 3·3 | 128 | — | <1 |
| A1/3 × DT2 | + | + | 2·5 | 64 | ND | |
| A1/3 × D1 | — | — | 1·5 | 16 | + + + | 17 |
| A1/3 × D1T | — | — | 1·4 | 16 | — | <1 |
| F1/3A4 (early passage) | — | — | 14 | 128 | — | 0 |
| F1/3A4 (>10 passage) | — | — | ND | 128 | + | 16 |
| F1/3A4 T1 | — | — | 1·8 | 256 | — | <1 |
| F1/3A4 T2 | — | — | 2·0 | 128 | — | 0 |
| BHK21-C13 Polyoma-transformed (pyT) | + | + | 8 | 128 | — | 1 |

* All tests were carried out on low passage (10 or less) cells except for F1/3A4, which was tested at higher passage number also. Tumour cell lines were established from tumours in hamsters, except A1/3 × DT2 and pyT, which were obtained from immunosuppressed mice. The parental cell lines used for inocula are given first, followed by cell lines established from the respective tumours.

† Increase in cell number after 4 days at 39°C when cells seeded at $10^3$ cells/cm². + represents at least a 5-fold increase.

‡ + denotes PE > 5%; — denotes PE < 0·1%.

§ Cells grown to confluency without medium change.

‖ Reciprocal of dilution of lectin giving 10% or less of cells agglutinated.

¶ The estimated intensity of labelling from radioautographs of SDS-polyacrylamide gels of samples of iodinated cells containing equal amounts of protein. The percentage of radioactivity in gel slices containing the 250K glycoprotein relative to the total radioactivity recovered from the resolving gel (excluding the radioactivity migrating as free iodine) is also shown.

presence of low Con A concentrations. At higher passage numbers (>10) the cultures maintain a high proportion of small cells with a rounded morphology, but increasing numbers of fibroblastic cells appear. Eventually the culture is dominated by fibroblastic cells. The growth properties and lectin agglutinability of cells recovered from such cultures are indistinguishable from the cells obtained at earlier passages.

Table I gives the tumorigenicity of various adenovirus-5-transformed BHK cells, compared to that of normal untransformed BHK cells, in adult Syrian hamsters. The normal BHK cells produced no tumours, even at the highest dose used ($10^6$ cells/animal) during the 6 months of observation. However, each of the transformant cell lines produced solid tumours, albeit at different rates (Table I). The tumours grew progressively to a palpable size within one month usually, and to a considerable diameter (4–5 cm). The small round-cell line, F1/3A4, which at early passage contained few fibroblastic cells, was apparently less tumorigenic than the same cell line at higher passage which had an appreciable proportion of cells with fibroblastic morphology, and was less tumorigenic than the other flat polyploid variants of the A1/3 × D series. In immunosuppressed (thymectomized, irradiated and reconstituted, $T^-B^+$) mice, normal BHK cells produced solid tumours (4/5 animals) when $10^5$ cells or more were inoculated s.c. Adenovirus transformant A1/3 × D was markedly more tumorigenic than normal BHK cells in this system. Inocula of $10^3$ and $10^4$ cells produced tumours (4/4 animals) at similar rates and of a similar size to polyoma-transformed BHK cells.

Tumour cell lines were established from growing tumours produced in hamsters by the variant cell lines A1/3 × D, A1/3 × D1, and F1/3A4 cells inoculated at early or late passages. The tumours were excized, dispersed with warm 0·25% trypsin and cultured in the medium used for parental cell lines. These tumours are designated A1/3 × DT1, A1/3 × D1T, F1/3A4T1 and F1/3A4T2 respectively (Table II). Each line was propagated serially *in vitro*, and after 3–5 passages stained positively (Meager *et al.*, 1975) for adenovirus T antigen. Microscopic examination determined that in the case of the flat polyploid cells A1/3 × D and A1/3 × D1 the morphology of the derived tumour cell lines reflected that of the particular cell line producing the tumour. However, cell lines derived from tumours produced by F1/A4 were always predominantly fibroblastic. The general growth characteristics of the tumour lines F1/3A4T1 and T2 were similar to those of the input cells (Table II) except that they did not grow out to the high saturation characteristic of the parental F1/3A4 line. The tumour cell lines A1/3 × DT1 and A1/3 × D1T grew to similar low saturation densities to the input cells, A1/3 × D and A1/3 × D1 respectively. The line A1/3 × D1T did not grow in agar suspension or 0·5% serum (Table II) whereas line A1/3 × DT1 grew well both in agar and low serum. In these characteristics the tumour cell lines resemble the input cells. A tumour line A1/3 × DT2, obtained from a tumour produced by inoculation of the flat

FIG. 1.—Lactoperoxidase-catalysed radio-iodination of (A) BHK21-C13 cells, (B) adenovirus-transformed variant cell line F1/3A4 iodinated at early passage number, and (C) a similar cell line at later passage number, showing reversion to a more fibroblastic morphology. The labelling and SDS-polyacrylamide-gel electrophoresis of the cell proteins are described in Meager *et al.* (1975). The slab gels were sliced into 2mm slices for counting γ radioactivity. Electrophoresis is from left to right.

polyploid cells Al/3×D into immunosuppressed mice, also closely resembled the morphology and growth characteristics of Al/3×D cells (Table II). The tumour cells harboured adenovirus T antigen and, furthermore, were sensitive to complement-mediated lysis in the presence of anti-BHK21-C13 serum, showing their origin.

The 250K glycoprotein species is present to varying extents in different adenovirus-5-transformed BHK cell lines (Meager *et al.*, 1975). Thus, Al/3×D and Al/3×Dl lines exhibit similar amounts of this glycoprotein to normal BHK cells (Fig. 1A) whereas in Fl/3A4 it appears to be absent or undetectable (Fig. 1B). However, after 10 or more serial passages the small round cells were completely replaced by fibroblastic cells, and when these "revertant" cells were examined (Fig. 1C) they showed iodination profiles more similar to normal BHK cells. In particular, a prominent 250K species appeared. The labelling profiles of tumour cell lines Fl/3A4Tl, Fl/3A4T2 and Al/3×DT, seemed to be very similar to one another (Fig. 2A–C). Each profile consisted of 2 major radioactive bonds, and none of the cell lines showed a major peak of radioactivity corresponding to a molecular weight of 250K.

Summarizing, no simple correlation between expression of 250K glycoprotein or other growth-related properties and tumorigenicity was apparent, in agreement with others (Berman, 1975; Glimelius *et al.*, 1975). The heterogeneous nature of transformants produced by adenoviruses, and the observed cell heterogeneity within any established cell line of adeno-transformant, make it quite possible for a number of highly tumorigenic variants to exist. Consistent with this was the observation that cell lines derived from tumours produced by the small round cell line, Fl/3A4 consisted of malignant fibroblastic cells. The similarities between the surface expression of glycoproteins of the various tumour cell lines, especially the very similar surface-labelling profiles of tumour

FIG. 2.—Lactoperoxidase surface-labelling of cells derived from hamster tumours induced by BHK21-C13 adenovirus-transformed variants. (A) Fl/3A4Tl cells; (B) Fl/3A4T2 cells; (C) Al/3×DIT cells.

cell lines derived from cells showing high amounts of the 250K glycoprotein (Al/3× Dl), compared to 250K-negative cells (*e.g.* Fl/3A4), may suggest selection, in the animals, of highly tumorigenic cells which all have similar cell surfaces, and in particular lack the iodinateable surface 250K glycoprotein. Since all the tumour cell lines are transplantable into fresh animals it may be reasonable to speculate that tumorigenicity and malignant growth potential are related to cell-surface 250K

glycoprotein expression (Chen *et al.*, 1976). Although the functions of this ubiquitous surface glycoprotein are unknown (Hynes, 1974; Vaheri *et al.*, 1976), it is commonly believed that a role in the formation or maintenance of a normal extracellular matrix which regulates orderly cell growth and organization is likely. A breakdown of the extracellular matrix resulting from loss of the surface glycoprotein may contribute to the disorderly growth and metastatic potential of transformed cells. It remains to be determined whether the apparent disappearance of the 250K surface glycoprotein is due to selection of a negative clone within the existing population of input cells, or due to epigenetic events taking place after inoculation into the host animal. In the latter case it would be expected that cells grown in culture for prolonged periods might gradually reacquire the surface 250K glycoprotein. This does not seem to be the case, however, suggesting that the loss of the 250K glycoprotein from BHK variants containing this glycoprotein before inoculation into animals is a regular event accompanying tumour formation. This does not, of course, prove clonal selection of a 250K-negative tumorigenic cell, but favours this conclusion.

### REFERENCES

Berman, L. D. (1975) Lack of Correlation between Growth Characteristics, Agglutinability by Plant Lectins and the Malignant Phenotype. *Int. J. Cancer*, **15**, 973.

Chen, L. B., Gallimore, P. H. & McDougall, J. K. (1976) Correlation between Tumour Induction and the Large External Transformation Sensitive Protein on the Cell Surface. *Proc. natn. Acad. Sci., U.S.A.*, **73**, 3570.

Glimelius, B., Nilsson, K., Ponten, J. (1975) Lectin Agglutinability of Non-neoplastic and Neoplastic Human Lymphoid Cells *In vitro*. *Int. J. Cancer*, **15**, 888.

Hynes, R. O. (1974) Role of Surface Alterations in Cell Transformation: the Importance of Proteases and Surface Proteins. *Cell*, **1**, 147.

Kao, F-T. & Harris, H. (1975) Lack of Correlation between Malignancy and Sensitivity to Killing by Concanavalin A. *J. natn. Cancer Inst.*, **54**, 767.

Meager, A., Nairn, R. & Hughes, R. C. (1975) Analysis of Transformed Cell Variants of BHK21 C13 Isolated as Survivors of Adenovirus Type 5. *Virology*, **68**, 41.

Nairn, R. & Hughes, R. C. (1976) Solubilization and Peptide Mapping of a Large External Glycoprotein Fraction Labelled by Lactoperoxidase-catalysed Iodination of Cultured Fibroblasts. *Biochem. Soc. Transactions*, **4**, 165.

Vaheri, A., Ruoslahti, E., Linder, E., Wartiovaara, J., Keski-Oja, J., Kuusela, P. & Saksela, O. (1976) Fibroblast Surface Antigen (SF): Molecular Properties, Distribution *In vitro* and *In vivo* and Altered Expression in Transformed Cells. *J. Supramolec. Structure*, **4**, 63.